# Telomere-related prognostic biomarkers for survival assessments in pancreatic cancer

**DOI:** 10.1038/s41598-023-37836-0

**Published:** 2023-06-30

**Authors:** Shengyang Chen, Shuiquan Hu, Baizhong Zhou, Bingbing Cheng, Hao Tong, Dongchao Su, Xiaoyong Li, Yanjun Chen, Genhao Zhang

**Affiliations:** 1 Department of Hepatobiliary Pancreatic Surgery, Zhengzhou University Fifth Affiliated Hospital, Kangfu Front Street 3#, Zhengzhou, 450052 China; 2 Department of Blood Transfusion, Zhengzhou University First Affiliated Hospital, Zhengzhou, China

**Keywords:** Cancer microenvironment, Gastrointestinal cancer, Tumour biomarkers

## Abstract

Human telomeres are linked to genetic instability and a higher risk of developing cancer. Therefore, to improve the dismal prognosis of pancreatic cancer patients, a thorough investigation of the association between telomere-related genes and pancreatic cancer is required. Combat from the R package “SVA” was performed to correct the batch effects between the TCGA-PAAD and GTEx datasets. After differentially expressed genes (DEGs) were assessed, we constructed a prognostic risk model through univariate Cox regression, LASSO-Cox regression, and multivariate Cox regression analysis. Data from the ICGC, GSE62452, GSE71729, and GSE78229 cohorts were used as test cohorts for validating the prognostic signature. The major impact of the signature on the tumor microenvironment and its response to immune checkpoint drugs was also evaluated. Finally, PAAD tissue microarrays were fabricated and immunohistochemistry was performed to explore the expression of this signature in clinical samples. After calculating 502 telomere-associated DEGs, we constructed a three-gene prognostic signature (DSG2, LDHA, and RACGAP1) that can be effectively applied to the prognostic classification of pancreatic cancer patients in multiple datasets, including TCGA, ICGC, GSE62452, GSE71729, and GSE78229 cohorts. In addition, we have screened a variety of tumor-sensitive drugs targeting this signature. Finally, we also found that protein levels of DSG2, LDHA, and RACGAP1 were upregulated in pancreatic cancer tissues compared to normal tissues by immunohistochemistry analysis. We established and validated a telomere gene-related prognostic signature for pancreatic cancer and confirmed the upregulation of DSG2, LDHA, and RACGAP1 expression in clinical samples, which may provide new ideas for individualized immunotherapy.

## Introduction

The purpose of telomeres, which are highly conserved nuclear protein structures made up of TTAGGG repeating base sequences and found at the ends of eukaryotic linear chromosomes, is to prevent the DNA damage response (DDR) from being activated and to ensure genomic integrity^1,2^. In fact, the length of telomeres becomes shorter each time a somatic cell divides because it is impossible for DNA polymerase to completely replicate the ends of linear chromosomes. When the length of telomeres shortens to a certain limit, the cell promotes spontaneous apoptotic or senescence mechanisms^3^. Although most human somatic cells do not display telomerase activity due to telomerase reverse transcriptase (TERT) gene suppression, it does not apply to cancer cells. Cancer cells' progressive telomere shortening activation can be avoided by activating the telomere maintenance mechanism (TMM)^4^, which consists of two main forms, telomerase activation and Alternative-lengthening of Telomere (ALT). Although telomerase-positive cancer cells can maintain their telomere length and achieve unlimited proliferation capacity by activating telomerase, their telomeres are usually dramatically shorter than those of normal cells, mainly due to the late activation of telomerase during tumorigenesis; as for ALT-dependent cancers, although telomeres are longer, there is greater heterogeneity^5^. Although ALT is relatively rare in cancer^6^, it is observed with high frequency in pancreatic neuroendocrine carcinoma^7^. Pancreatic neuroendocrine carcinoma accounts for only 1–2% of pancreatic tumors, but the unique genetic profile that distinguishes it from gland-dependent pancreatic tumors warrants further in-depth analysis, which will help to improve the evaluation of risk assessment. Due to the small number, pancreatic neuroendocrine tumors were not included in this study. Activated telomerase keeps telomeres in equilibrium over time and reduces genomic instability to tolerable levels, thereby enabling the near-immortality of tumor cells. In most types of tumors in the TCGA database, longer telomeres are observed in normal adjacent tissues than in telomerase-positive tumors^8^. Therefore, the study of the correlation between tumors and telomeres has been a hot topic.

Telomeres play a two-sided role in the development of tumors. On the one hand, telomere shortening can inhibit cell proliferation and hinder tumor growth, while on the other hand, telomere shortening can also promote cancer development by causing widespread genomic instability. Although telomerase-activated cancers can lengthen telomeres, most tumors have shorter telomere lengths than normal tissue. Reduced DKC1 expression induces telomere-associated senescence and apoptosis in lung adenocarcinoma^9^. In addition, telomere shortening was a potential risk factor for pancreatic cancer^10^. These all suggest a potential use of telomere length as a predictive biomarker for tumors.

Considering that previous studies have focused on telomere length in pancreatic cancer and its role in pancreatic cancer prognosis, there is still a lack of relevant studies on the relationship between telomere-related genes and pancreatic cancer prognosis. In the present study, we constructed and validated a prognostic signature of pancreatic cancer based on the telomere-related genes, and also evaluated the underlying value of this signature in the selection of therapeutic drugs.

## Methods

### Acquisition of telomere-relevant genes and publicly available data

A total of 2086 telomere-relevant genes were downloaded from the TelNet database^11^. RNA sequencing (RNA-seq) data of The Cancer Genome Atlas-PAAD (TCGA-PAAD) cohort and the GTEx database obtained from the UCSC Xena project (https://xenabrowser.net) were combined as a discovery cohort for screening differentially expressed genes (DEGs) and prognostic model construction. After normalizing the RNA-seq data using a log2 (FPKM + 1) transformation, Combat from the R package “SVA” was performed to correct the batch effects between the two datasets. Samples with no complete survival data or survival time of less than 1 month were excluded. DEGs were screened out using an adjusted *P* value of less than 0.05 and |logFC| ≥ 1 as cutoff criteria. Data of the PACA-CN cohort was obtained from the ICGC database (https://dcc.icgc.org/), and GSE62452, GSE71729, and GSE78229 cohorts from the gene expression omnibus (GEO) database (https://www.ncbi.nlm.nih.gov/geo/) served as the test cohort.

### Prognostic model construction and validation

The telomere-relevant DEGs were assessed to construct a prognostic risk model through univariate Cox regression, LASSO-Cox regression, and multivariate Cox regression as we previously reported^12^. Risk score = ∑iCoefficient (mRNAi)*Expression (mRNAi). We first divided the pancreatic cancer patients in the five datasets mentioned above into two subgroups according to a range of ideal risk scores determined by the R “survminer” package and then performed a Kaplan–Meier survival check to see if there was a difference in survival rates between the two groups. The ROC check was used to assess the prognostic effect of the model across the five datasets. Cox risk regression models were used to assess whether the prognostic model was an independent prognostic factor for patients with pancreatic cancer.

### Characterization of functional analysis

To investigate substantially changed HALLMARK, GO, and KEGG items, the Metascape database was used^13,14^.

### Immune cell infiltration and immune checkpoint gene analysis

Based on the expression patterns of multiple immune and stromal cell genes in tumor samples from pancreatic cancer patients, ESTIMATE was used to estimate the stromal and immune fractions of the tumor tissue^15^. A higher immune score represents a higher proportion of immune cell infiltration, a higher stromal score represents a higher proportion of stromal cell infiltration, and a higher ESTIMATE score represents a lower purity of tumor cells. Additionally, the abundance ratio of tumor-infiltrating immune cells (TIICs) was measured using the CIBERSORT^16^, xCELL^17^, MCPcounter^18^, and TIMER^19^ databases to more thoroughly examine the distribution variations of TIICs in the pancreatic cancer TME. Last but not least, we evaluated the variations in gene expression across distinct immune checkpoint subgroups for PD1, PD-L1, CD276, CTLA4, LAG3, CXCR4, IL1A, IL6, TGFB1, TNFRSF4, TNFRSF9, and PD-L2.

### Potential therapeutic drug sensitivity analysis

We first downloaded NCI-60 compound activity data and RNA-seq expression profiles from the CallMiner database^20^ and selected Food and Drug Administration (FDA)-approved drugs or drugs from clinical trials for subsequent analysis. Based on the differential expression of the three genes in the prognostic model, we assessed the scores of each sample and analyzed the correlation between drugs and scores. Drugs were deemed tumor-sensitive if their adjusted *P* values were less than 0.001 and their Pearson correlation coefficients were greater than 0.3. Then, we contrasted the variations in half maximum inhibitory concentration (IC50) of tumor-sensitive medicines amongst various scores groupings. The results were declared statistically significant when the *P* value was less than 0.05.

### Immunohistochemistry (IHC) evaluation

A validation cohort was created using 30 samples of pancreatic cancer and associated paracancerous tissues that had been validated by normal pathological investigation from our pathology department. All samples have comprehensive clinical information. Following serial sectioning of the tissue wax blocks, sample sections were collected and kept in a 4 °C freezer for later use. We subsequently performed IHC assays on formalin-fixed de-paraffinized sections using DSG2 (Abcam, ab150372), LDHA (Abcam, ab52488), and RACGAP1 (Abcam, ab134972) antibodies and took images with microscopy as described previously^21^.

### Statistical analysis

Continuously distributed numerical data were evaluated using the grouped t-test or Mann–Withney–Wilcoxon test, whilst the qualitative data were examined using the chi-square test. Using the survival and survminer package, Kaplan–Meier survival analysis was done to determine overall survival rates between groups. Using the ‘timeROC’ package, receiver operating characteristic (ROC) curves were created to determine the area under the curve (AUC) values at 1, 2, and 3 years. To determine if the signature was an independent prognostic predictor, univariate and multivariate Cox regression analyses were carried out. Based on the findings, a nomogram was created using the RMS package. The Pearson or Spearman correlation test was used to do the correlation study. The results were declared statistically significant when the *P* value was less than 0.05.

### Ethics approval and consent to participate

This study was approved by the Ethics Committees of Zhengzhou University. Written informed consent was obtained from all patients. All methods were performed following the relevant guidelines and regulations.

## Results

### Identification of telomere-related DEGs and roles of telomere-related genes

**Figure 1:**
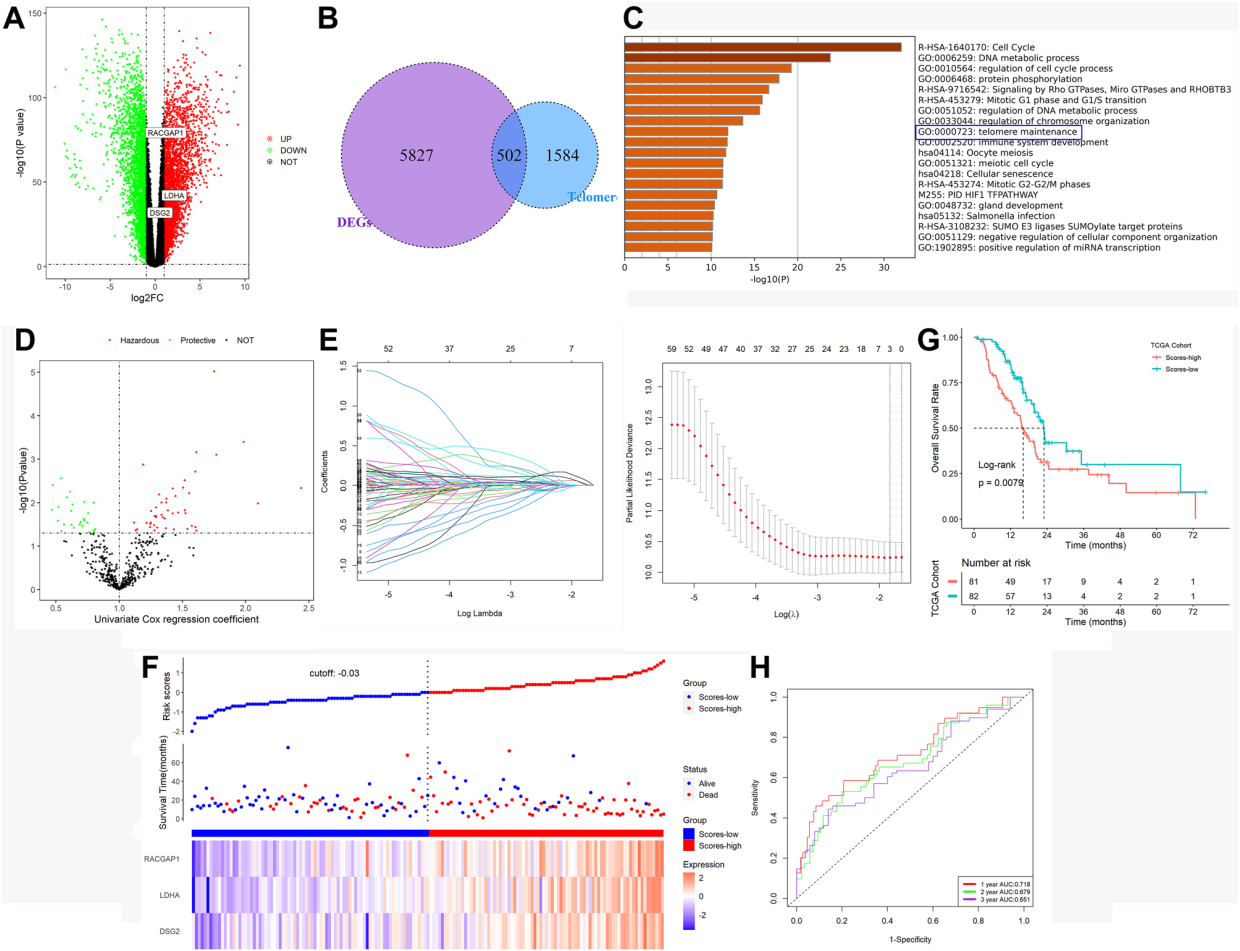
Identification of telomere-related DEGs and establishment of telomere-related subtypes (**A**) DEGs between the 171 normal pancreatic tissues and 178 pancreatic cancer tissues. (**B**) 502 overlapping genes were identified as telomere-related DEGs. (**C**) GSEA enrichment results of these telomere-related DEGs. (**D**) 72 of the 502 telomere-related DEGs were calculated as prognostic genes. (**E**) LASSO-Cox regression analysis. (**F**) The scores of patients and their distribution. (**G**) Patients with higher risk ratings exhibited noticeably lower survival outcomes. (**H**) ROC analysis.

After batch effect removal, we screened out 6329 DEGs between the 171 normal pancreatic tissues and 178 pancreatic cancer tissues (Fig. 1A). Then, 502 telomere-related DEGs were identified (Fig. 1B). In addition, these telomere-associated DEGs could distinguish well between normal and pancreatic cancer tissue samples (Supplementary Fig. S1), suggesting that telomere-related genes contributed to tumor heterogeneity and may have a key role in the tumorigenesis of pancreatic cancer. According to the results of Gene Set Enrichment Analysis (GSEA), these genes are mainly enriched in biological processes related to the cell cycle and telomere homeostasis (Fig. 1C). Overall, we obtained 502 telomere-associated DEGs for subsequent analysis.

### Establishment of telomere-related subtypes

**Figure 2:**
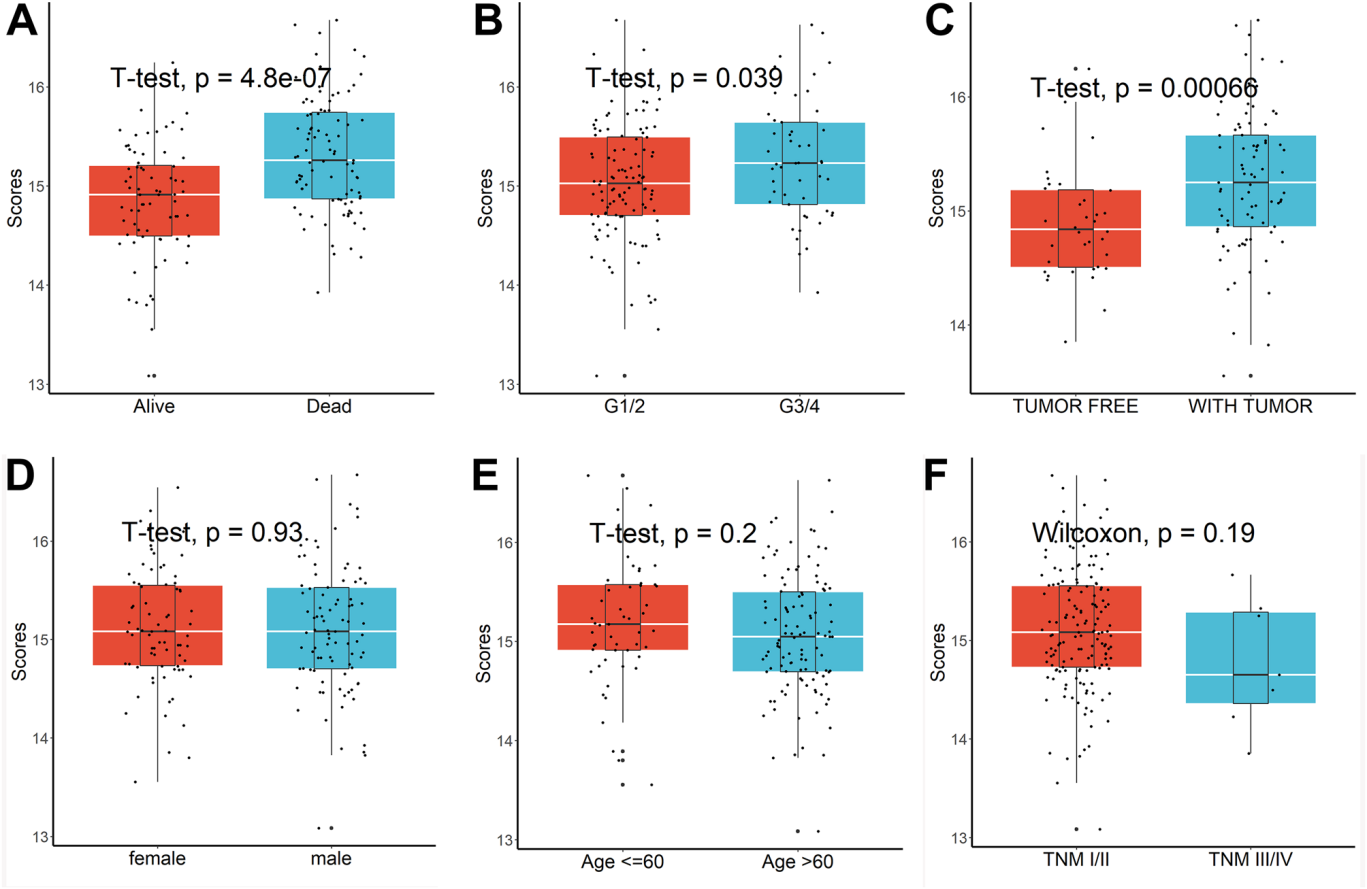
Patient death (**A**), advanced grades (**B**), and relapses (**C**) were all linked to higher risk scores, while gender (**D**), age (**E**), and TNM stage (**F**) were not associated with risk scores.

According to the univariate Cox regression analysis, 72 of the 502 telomere-related DEGs were calculated as prognostic genes (Fig. 1D). DEGs with increased expression in tumors are risk genes while DEGs with down-regulated expression in tumors are protective genes. These prognostic DEGs were explored through the LASSO-Cox regression and multivariate Cox proportional regression (Fig. 1E). Three genes (DSG2, LDHA, and RACGAP1) were finally screened for the construction of prognostic models. Score = (0.3045195 × DSG2) + (0.4734231 × RACGAP1) + (0.4349522 × LDHA). The scores of patients and their distribution were shown in Fig. 1F. We found that the score correlated with most telomere-related genes (Supplementary Table S1). Patients with higher risk ratings exhibited noticeably lower survival outcomes when the optimal risk score threshold was employed to classify patients into high- or low-risk groups (Fig. 1G). With AUCs at 1-, 2-, and 3-year of 0.718, 0.679, and 0.651, this classifier displayed high predictive performance (Fig. 1H). In addition, patient death (Fig. 2A), advanced grades (Fig. 2B), and relapses (Fig. 2C) were all linked to higher scores, while gender, age, and TNM stage were not associated with scores (Fig. 2D–F). Finally, univariable and multivariate Cox regression analysis showed that this signature may be employed as a standalone prognostic factor for individuals with pancreatic cancer (univariable HR = 2.718, 95%CI 1.822–4.056, *P* = 9.7e−07; multivariate HR = 2.445, 95%CI 1.459–4.097, *P* = 0.0006). Overall, we constructed a three-telomere gene-related model that can be used to accurately predict patient prognosis.

### Verification of the signature in multiple additional datasets

**Figure 3:**
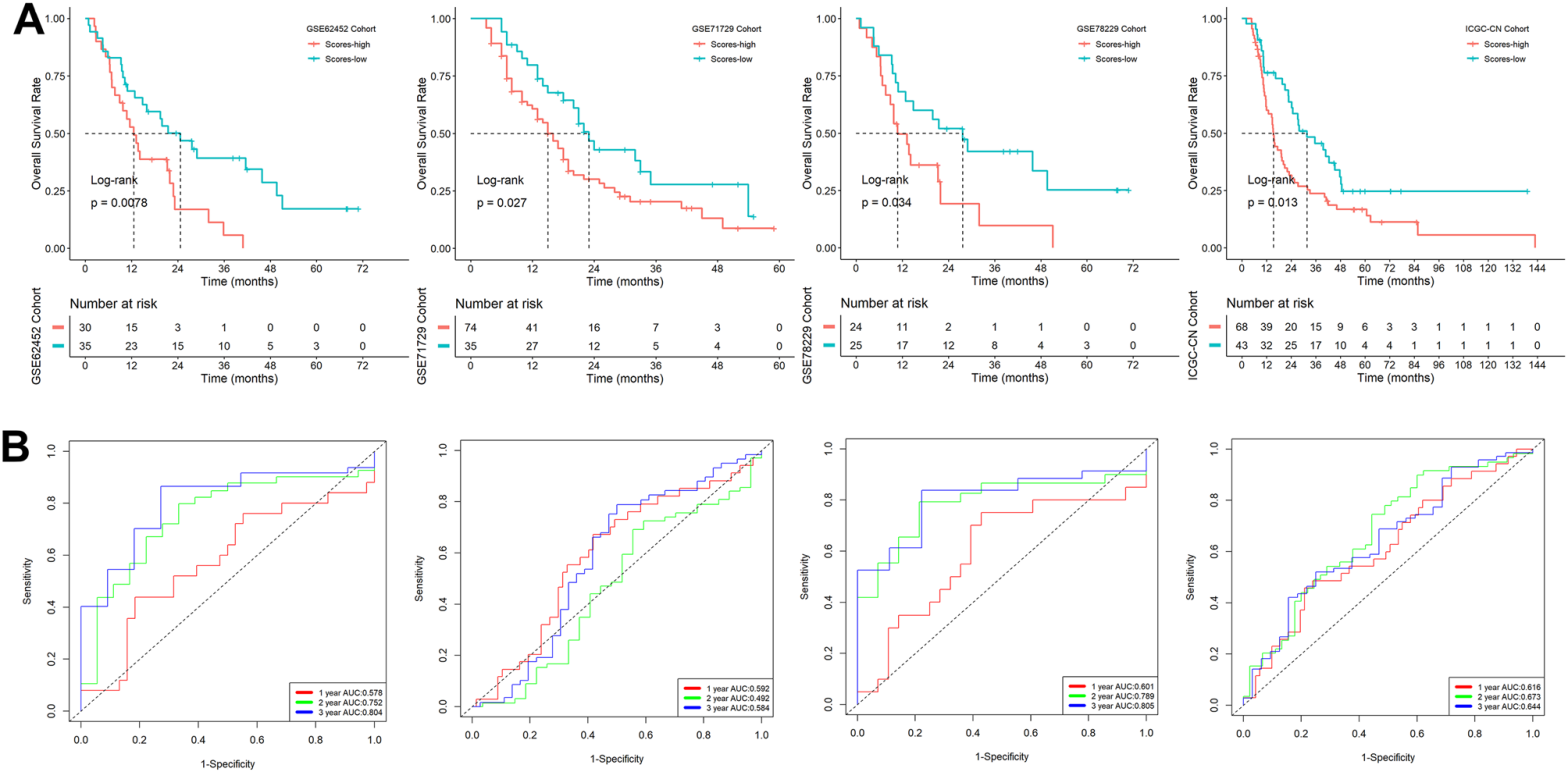
Verification of the signature in multiple additional datasets. (**A**) Kaplan–Meier survival analysis. (**B**) ROC analysis.

We calculated risk scores for patients in the ICGC, GSE62452, GSE71729, and GSE78229 datasets using the same formula and validated this signature in these patients. Patients with greater risk scores were significantly related to having lower OS rates, according to Kaplan–Meier survival analysis (Fig. 3A), and according to ROC analysis, this signature exhibited good prognostic performance (Fig. 3B). Overall, the model we constructed also accurately predicted the prognosis of patients in the validation set.

### Characterization of genomic variation and functional analysis

**Figure 4:**
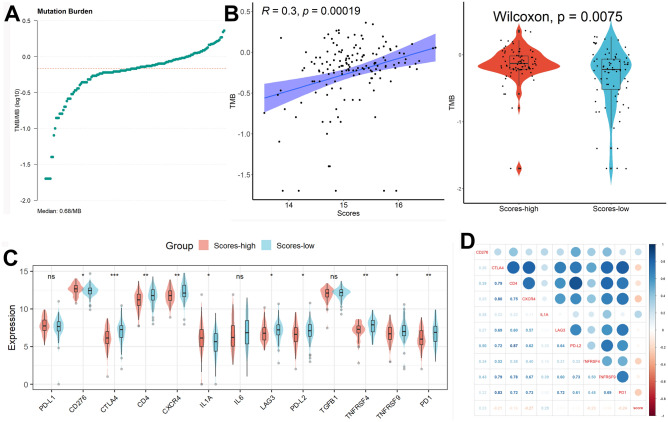
Characterization of ICIs analysis. (**A**) The tumor mutational burden (TMB) value of each pancreatic cancer patient. (**B**) The TMB value was greater in patients in the high-risk group and the risk score was strongly positively linked with the TMB value. (**C**) Patients with lower scores had higher levels of the genes CTLA4, CD4, CXCR4, LL1A, TNFRSF4, and PD1 than patients with higher scores. (**D**) Scores were strongly correlated with the expression levels of CTLA4, CD4, CXCR4, and LL1A.

According to the GSEA results, the major enrichment of differential genes between high and low-scoring subgroups for HALLMARK entries was “HALLMARK ALLOGRAFT REJECTION”, “HALLMARK E2F TARGETS”, “HALLMARK G2M CHECKPOINT”, “HALLMARK GLYCOLYSIS”, and “HALLMARK MITOTIC SPINDLE”, for GO entries was “GOBP ACTIVATION OF IMMUNE RESPONSE”, “GOBP ADAPTIVE IMMUNE RESPONSE”, “GOBP ANTIGEN RECEPTOR MEDIATED SIGNALING PATHWAY”, and “GOBP ATTACHMENT OF SPINDLE MICROTUBULES TO KINETOCHORE”, and for KEGG entries was “KEGG CHEMOKINE SIGNALING PATHWAY”, “KEGG CYTOKINE CYTOKINE RECEPTOR INTERACTION”, “KEGG NEUROACTIVE LIGAND RECEPTOR INTERACTION”, “KEGG CELL CYCLE”, and “KEGG CELL ADHESION MOLECULES CAMS” (Supplementary Fig. S2). Overall, telomere scores may be involved in the prognostic regulation of pancreatic cancer patients by influencing the cell cycle of tumor cells, the body's immune response, and chemotherapy-related biological processes. After analyzing the tumor mutational burden (TMB) value of each pancreatic cancer patient (Fig. 4A), we discovered that the TMB value was greater in patients in the high-risk group and that the risk score was strongly positively linked with the TMB value (Fig. 4B), suggesting that immunotherapy might be less effective in patients with higher scores. Additionally, we discovered that patients with lower scores had higher levels of the genes CTLA4, CD4, CXCR4, LAG3, PD-L2, TNFRSF4, TNFRSF9, and PD1 than patients with higher scores (Fig. 4C), and scores were strongly correlated with the expression levels of CTLA4, CXCR4, TNFRSF4, and PD1 (Fig. 4D). By activating immune checkpoints and inhibiting the presentation of antigens to T cells, tumor cells can escape surveillance and survive by blocking the process of antigen presentation in the tumor immune microenvironment, thereby inhibiting the immune function of T cells. The immune checkpoint inhibitor relieves this inhibition and allows the immune cells to be reactivated to work against the cancer cells. Due to the high expression of immune checkpoint genes in low-scoring patients, these ICIs may be more beneficial for patients with lower scores.

### Correlation analysis of the signature and TME

**Figure 5:**
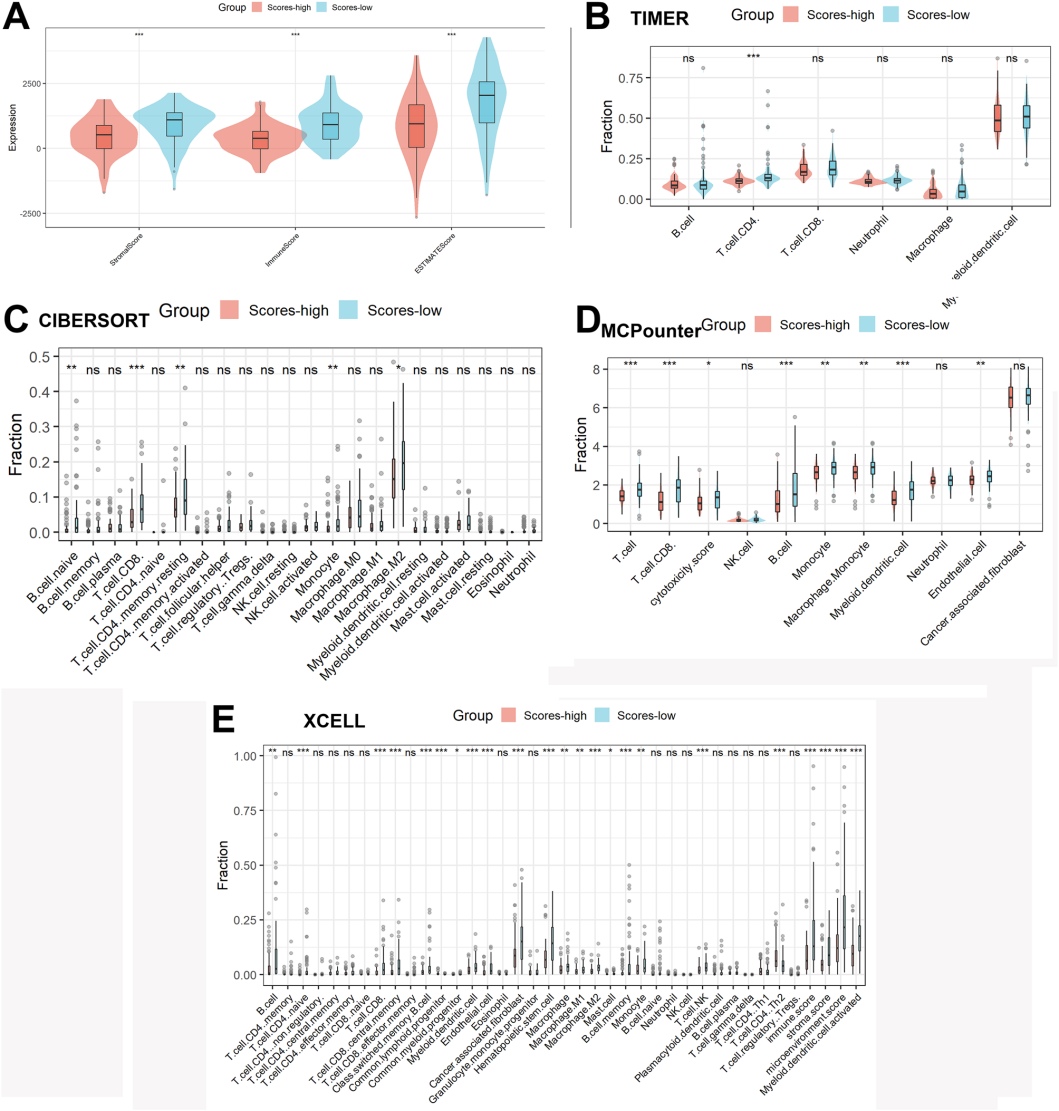
Correlation analysis of the signature and TME. (**A**) In comparison to patients with lower scores, patients with higher scores had lower stromal, immune, and estimate scores. Immune cell infiltration analysis by the TIMER (**B**), CIBERSORT (**C**), MCPcounter (**D**), and xCELL (**E**).

Figure 5A demonstrates that in comparison to patients with lower risk scores, patients with higher risk scores had lower stromal, immune, and estimate scores. Additionally, as seen in Fig. 5B, according to the TIMER database, patients with higher risk scores had lower abundance levels of CD4 T cells when compared to patients with lower risk scores. As seen in Fig. 5C, according to the CIBERSORT, patients with higher risk scores had lower abundance levels of naïve B cell, CD8 T cell, resting CD4 T cell, monocyte, and M2 macrophage when compared to patients with lower risk scores. As seen in Fig. 5D, according to the MCPcounter database, patients with higher risk scores had lower abundance levels of T cell, CD8 T cell, B cell, monocyte, endothelial cell, myeloid dendritic cell, and macrophage monocyte when compared to patients with lower risk scores. As seen in Fig. 5E, according to the xCELL database, patients with higher risk scores had lower abundance levels of B cell, naïve CD4 T cell, CD8 T cell, central memory CD8 T cell, class-switched memory B cell, common lymphoid progenitor, myeloid dendritic cell, endothelial cell, cancer-associated fibroblast, hematopoietic stem cell, macrophage, macrophage M1, macrophage M2, memory B cell, monocyte, NK T cell, Th1 CD4 T cell, and activated myeloid dendritic cell when compared with patients with lower risk scores. When the four algorithms described above are considered together, patients with lower risk scores possessed a higher number of immune cell infiltrates in TME, as shown in Supplementary Table S2, CD4 T cell was the common differential immune cell.

### Construction of a nomogram model

To test the efficacy of this signature's coefficient prediction, a nomogram model was created. The outcomes demonstrated that the nomogram with a concordance index (C-index) of 0.733 may assist in the creation of a precise quantitative technique for forecasting the 1-, 2-, and 3-year survival rates (Supplementary Fig. S3A). The projected and actual likelihood of 1-, 2-, and 3-year survival rates' overlap on the calibration curves revealed good agreement (Supplementary Fig. S3B). Overall, the nomogram can be used for clinician management of patients.

### Potential therapeutic drug sensitivity analysis

**Figure 6:**
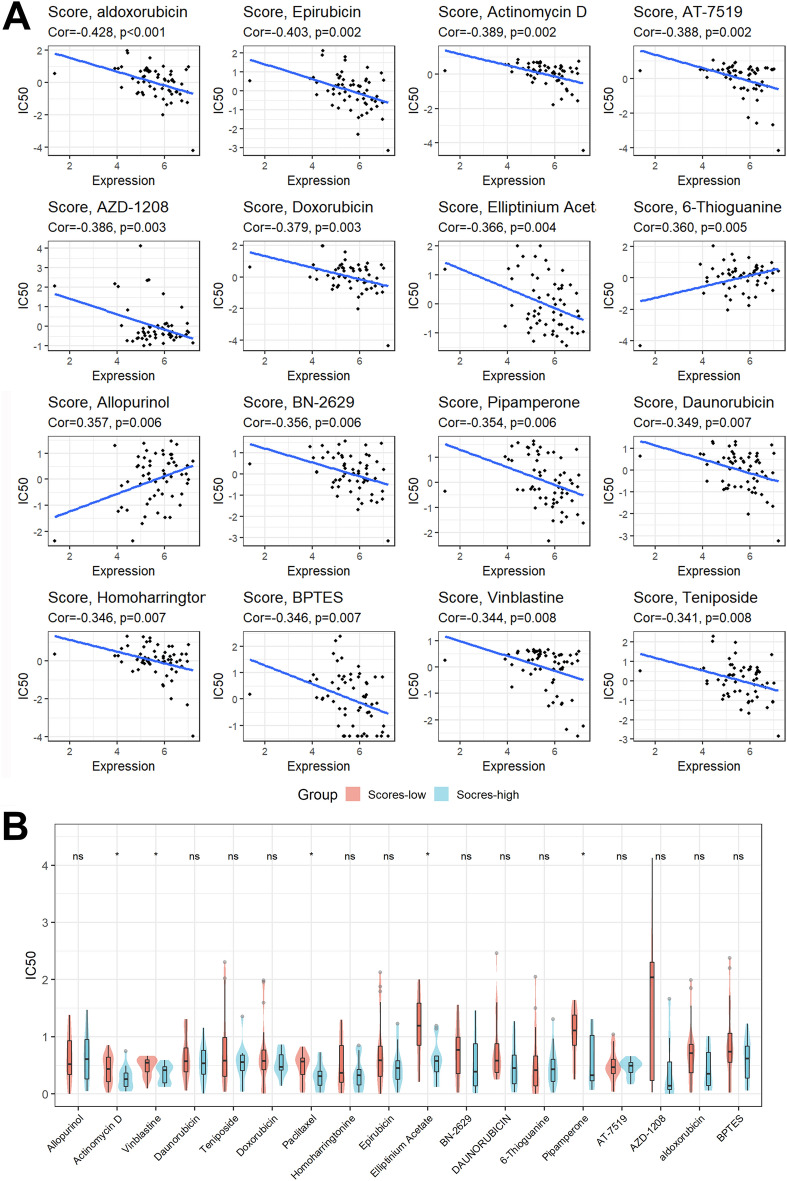
Potential therapeutic drug sensitivity analysis. (**A**) Top 16 most important tumor-sensitive drugs. (**B**) Differential analysis of the IC50 of tumor-sensitive drugs.

We found 18 tumor-sensitive drugs (Supplementary Table S3), and the top 16 most significant tumor-sensitive drugs were shown in Fig. 6A. Among the 18 tumor-sensitive drugs, as seen in Fig. 6B, we also found that patients with higher scores had a lower IC50 for Actinomycin D, Vinblastine, Paclitaxel, Elliptinium Acetate, and Pipamperone, implying that patients with higher scores were more sensitive to these drugs.

### Expression levels of DSG2, LDHA, and RACGAP1

**Figure 7:**
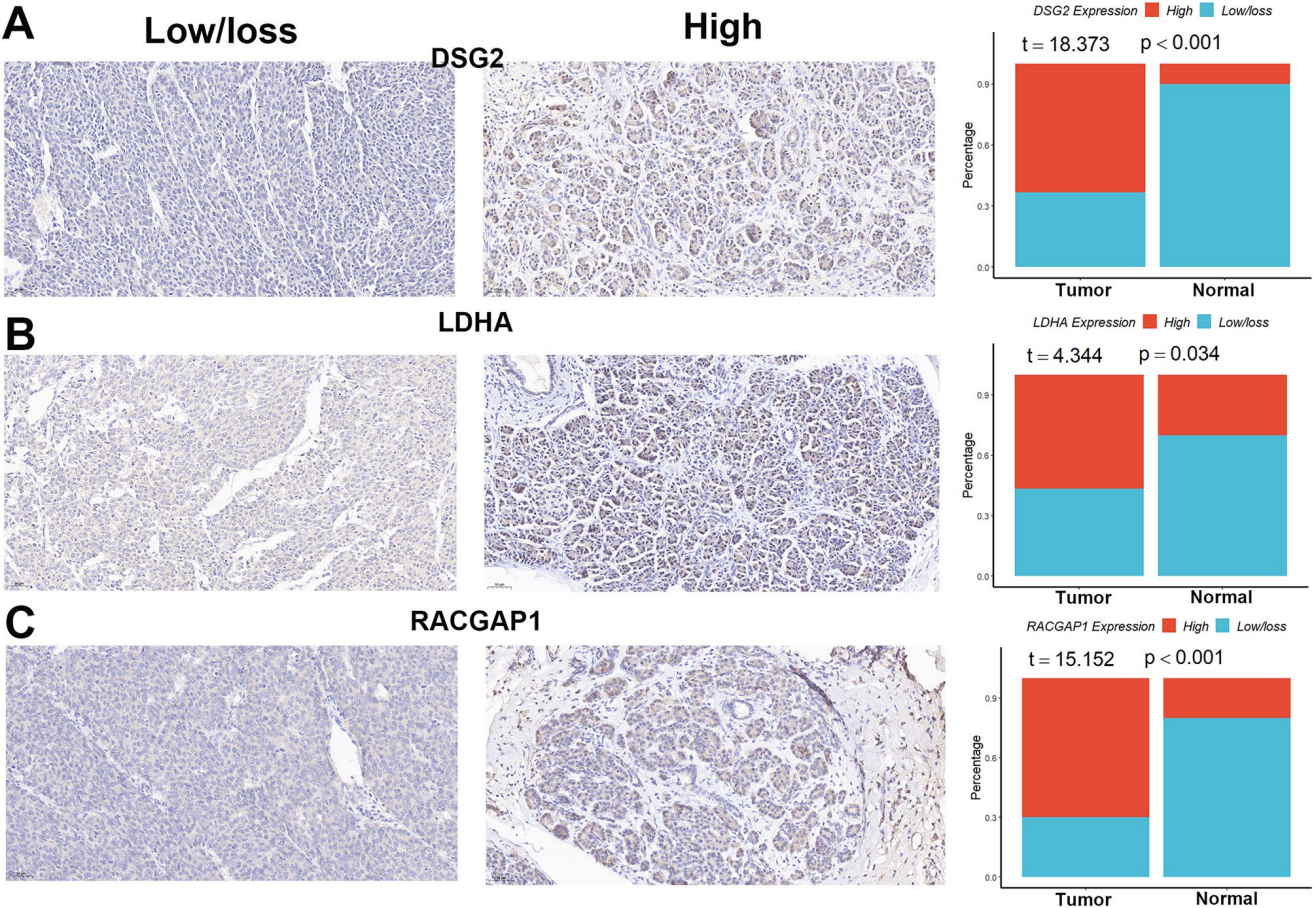
Protein expression levels and percentage of positivity for the three genes in clinical samples. (**A**) DSG2. (**B**) LDHA. (**C**) RACGAP1.

We first explored the expression levels of the three genes in the GEPIA database^22^. We found that all three genes were up-regulated in pancreatic cancer tissues compared to normal tissues (Supplementary Fig. S4A). We also found that high levels of DSG2, LDHA, and RACGAP1 predicted poorer overall (Supplementary Fig. S4B) and disease-free survival in pancreatic cancer patients (Supplementary Fig. S4C), although these three genes did not all show good performance in the four validation datasets (Supplementary Fig. S5A–D). We then explored the protein expression of DSG2, LDHA, and RACGAP1 in normal pancreatic and pancreatic cancer tissues in the human protein atlas database (HPA)^23^, and found that all of them showed differential expression (Supplementary Fig. S6). Finally, we examined the protein levels of DSG2, LDHA, and RACGAP1 in clinical samples using IHC techniques. According to Figs. 7A–C, pancreatic cancer tissues had higher protein levels of all three genes than normal tissues, which was in line with the bioinformatics study previously mentioned.

## Discussion

In the development of pancreatic cancer, telomeres are crucial^24^. Telomerase treatment was very useful for treating pancreatic cancer patients since telomere shortening and telomerase activation were common in human pancreatic cancer^25–30^. Because telomerase was repressed in the vast majority of human somatic cells, an immunotherapy that targeted tumor cell telomerase had no negative impact on normal cells. Furthermore, telomerase activity was only present in small subpopulations of normal human cells, such as stem/progenitor cells, activated lymphocytes, and other highly proliferative cells, but these cells had telomere lengths that were significantly longer than those of cancer cells, suggesting that tumor cells would respond more readily to telomerase inhibition therapy^31^. As a result, we may destroy telomeres in tumor cells before harm is done to telomerase-positive, healthy human cells. Notably, low residual levels of telomerase activity before telomerase function is eliminated may affect the efficacy of telomerase-targeted therapies^5^.

Desmoglein-2 (DSG2) is a cell adhesion protein belonging to the calmodulin superfamily that plays an important regulatory role in the functional development of cardiomyocytes^32^. DSG2 is also involved in the regulation of telomerase activity^8^. Although many studies have demonstrated that abnormal expression of DSG2 is associated with tumorigenesis^33^, its role in tumors is still controversial. The poor prognosis of patients with high expression of DSG2 in cutaneous squamous cell carcinoma and cervical cancer was correlated^34–36^. Downmodulation of DSG2 significantly inhibited the proliferation of colon cancer cells and the progression of non-small cell lung cancer^37,38^. In contrast, low expression of DSG2 was associated with shorter survival times in patients with gastric and prostate cancers^39,40^. In addition, reduced DSG2 expression also promotes lymph node metastasis in breast cancer^41^. Interestingly, although DSG2 was previously reported to be minimally expressed in pancreatic cancer^42^, in our study we found that DSG2 was highly expressed in pancreatic cancer tissues compared to normal pancreatic tissues and that high DSG2 expression was strongly associated with poor prognosis. This phenomenon may be caused by tumor heterogeneity in pancreatic cancer patients. Lactate dehydrogenase A (LDHA) is involved in the regulation of telomere signaling by PCA/BiFC (protein complementation assay/bimolecular fluorescence complementation) assay and co-precipitation experiments^43^. LDHA is also an important component of the glycolytic pathway and plays a key role in tumorigenesis^44^. Hypoxia-induced abnormal LDHA expression regulates the progression of gastric cancer^45^. Platinum complexes inhibit the metabolism and migration of triple-negative breast cancer cells by targeting LDHA^46^. Erythropoietin L-2HG can be produced by LDHA in hypoxic settings to maintain the proper balance of pancreatic cancer stem cell differentiation^47^. Reducing LDHA expression significantly increased the sensitivity of cancer cells to gemcitabine and inhibited tumor progression^48^. In our study, we found that LDHA can be used as a telomere-related gene to accurately predict the prognosis of pancreatic cancer patients. Rac GTPase-activating protein 1 (RACGAP1) is included in the TelNet database as a yeast homolog, but direct experimental validation is lacking, even though we have found RACGAP1 to be associated with telomere maintenance through GSEA (Supplementary Fig. S7). RACGAP1 is also a star molecule associated with poor tumor prognosis and is involved in the development and progression of many tumors. RACGAP1 can be involved in the regulation of breast cancer metastasis by targeting ECT2^49^. By controlling CDC25C, RACGAP1 encourages the growth of cervical cancer cells^50^. RACGAP1 deletion has also been associated with mitotic mutations in esophageal squamous cell carcinoma^51^. When creating gene models for metastasis that forecast overall survival for patients with pancreatic ductal adenocarcinoma, RACGAP1 can be used as a predictor^52^. In our study, we found that RACGAP1 can be used as a telomere-related gene to accurately predict the prognosis of pancreatic cancer patients.

A growing body of research indicates that TME contributes significantly to the development and spread of pancreatic cancer. TME contains a range of cell types that can be implicated in tumor development, growth, and migration, including immune cells, stromal cells, endothelial cells, complicated cytokine secretion, and fibroblasts^53^. When the outcomes of the four algorithms TIMER, CIBERSORT, XCELL, and MCPcounter were taken into account collectively, CD4+ T cells emerged as the distinct immune cells between the high- and low-risk subgroups, indicating that the telomere-related signature may modulate CD4+ T cell infiltration to worsen the prognosis of pancreatic cancer. Excessive infiltration of cancer-associated fibroblasts (CAFs) in TME is associated with poor survival in pancreatic cancer patients and can also induce the infamous clinical treatment resistance^54^. In addition, CAFs expressing MHC class II in TME have been reported to present antigens to CD4+ T cells and modulate immune responses in pancreatic tumors^55^. Considering that complex TME can induce resistance of pancreatic cancer cells to immune checkpoint inhibitors (ICIs) and thus affect the efficacy of immunotherapy in patients^56^, we also analyzed the variation in expression levels of multiple immune checkpoint genes among high- and low-risk subgroups. We discovered that patients with lower scores had higher levels of the genes CTLA4, CD4, CXCR4, TNFRSF4, and PD1 than patients with higher scores, and scores were strongly correlated with the expression levels of CTLA4, CXCR4, TNFRSF4, and PD1, suggesting that these ICIs may be more beneficial for patients with lower scores. Interestingly, Nobuhiko has reported that telomerase-specific cleavage adenovirus can be combined with ICIs to treat tumors^30^, so ICIs combined with specific cleavage adenovirus constructed based on telomere-related genes for improving the prognosis of pancreatic cancer patients will have a good prospect in the future.

Finally, we also analyzed the tumor-sensitive drugs targeting this signature through the CellMiner database. We identified a total of 18 tumor-sensitive drugs targeting this signature, with the IC50 for Actinomycin D, Vinblastine, Paclitaxel, Elliptinium Acetate, and Pipamperone being lower in patients with higher scores, implying that patients with higher scores are more sensitive to these drugs. Actinomycin-D (ActD) is a peptide antibiotic that can limit RNA and protein synthesis by inhibiting RNA polymerase II and can be used in conjunction with dimethylamine monophenol lactone to treat human pancreatic cancer cells^57^. A variety of Vinblastine-based bioactive drugs can inhibit the proliferation of tumor cells and shine in the treatment of pancreatic cancer^58,59^. Paclitaxel can be combined with gemcitabine to treat patients with metastatic pancreatic cancer and improve their overall survival^60–62^. As for Elliptinium Acetate and Pipamperone, their use in the treatment of pancreatic cancer patients has not yet been reported and deserves further attention in the future.

Our manuscript still has some highlights, even though other comparable publications have been published employing built characteristics to predict the prognosis of pancreatic cancer patients. First, we examined the patient's prognosis for pancreatic cancer for the first time using telomere-related genes. Second, we successfully confirmed the signature using several publicly available datasets. Finally, we examined clinical tissue samples to confirm the protein levels of the genes responsible for the composition of the signature. Of course, there are certain restrictions on our study. To assess this signature, future large multicenter randomized controlled investigations are required. In the future, further in vivo and in vitro research is required to further investigate the expression, prognostic predictive relevance, and particular mechanisms of these three genes in pancreatic cancer.

## Conclusion

We established and validated telomere gene-related prognostic features in pancreatic cancer and confirmed the upregulation of DSG2, LDHA, and RACGAP1 expression in clinical samples, which may provide new ideas for individualized immunotherapy targeting telomere genes.

## Supplementary Information

Supplementary Information.

## Data Availability

The datasets used and/or analyzed during the current study (including TCGA-PAAD, GTEx, ICGC-PACA-CN, GSE62452, GSE71729, and GSE78229 cohorts) are available from the corresponding author upon reasonable request.

## Abbreviations

DEGs: Differentially expressed genes
TME: Tumor microenvironment
IHC: Immunohistochemical staining
DDR: DNA damage response
TERT: Telomerase reverse transcriptase
TMM: Telomere maintenance mechanism
ALT: Alternative-lengthening of Telomere
RNA-seq: RNA sequencing
GSEA: Gene set enrichment analysis
FDA: Food and Drug Administration
IC50: Half maximum inhibitory concentration
ROC: Receiver operating characteristic curves
TMB: Tumor mutational burden
CCLE: Cancer cell line encyclopedia
HPA: Human protein atlas
DSG2: Desmoglein-2
LDHA: Lactate dehydrogenase A
RACGAP1: Rac GTPase-activating protein 1
ICIs: Immune checkpoint inhibitors
ActD: Actinomycin-D
