# Supplementary material for: Telomere-related prognostic biomarkers for survival assessments in pancreatic cancer
Source: Sci Rep. 2023 Jun 30;13:10586. doi: 10.1038/s41598-023-37836-0 (PMC10313686; doi:10.1038/s41598-023-37836-0)
Supplement: Supplementary file 1 — Supplementary Information. [file 41598_2023_37836_MOESM1_ESM.docx]

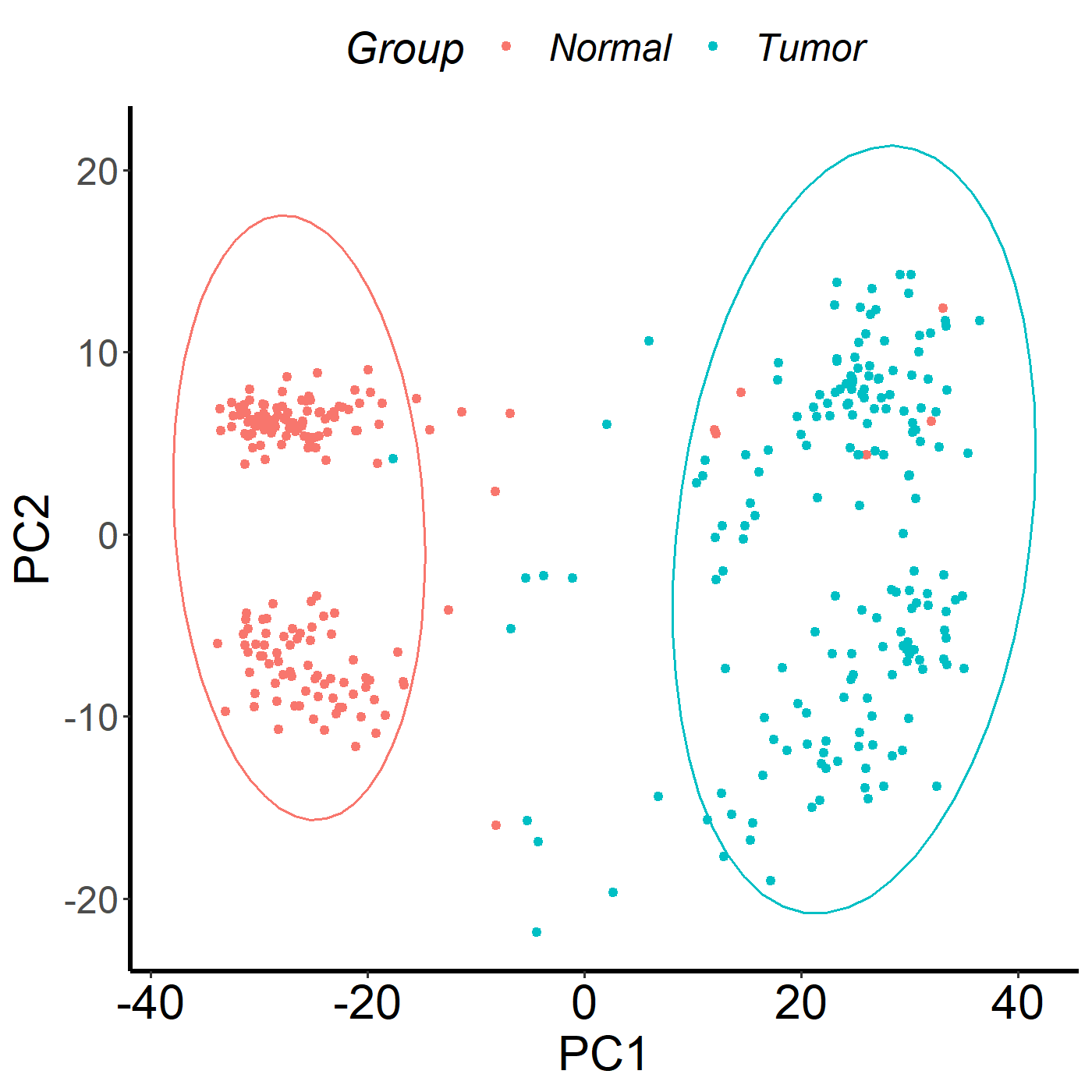


Figure S1 The distribution of all samples was analyzed using principal component analysis (PCA).


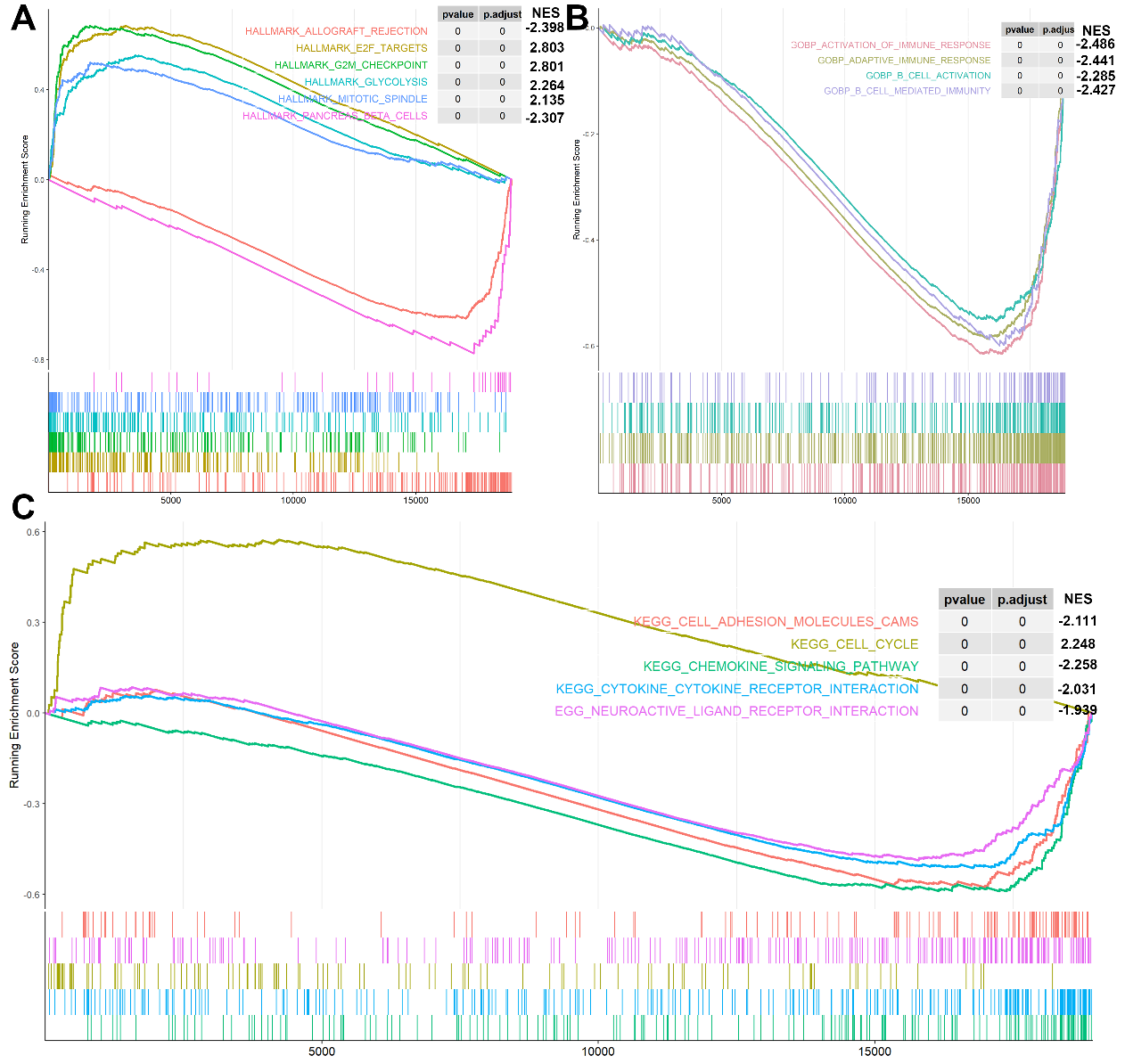


Figure S2 Affected HALLMARK (A), GO (B), and KEGG (C) items in the signature.


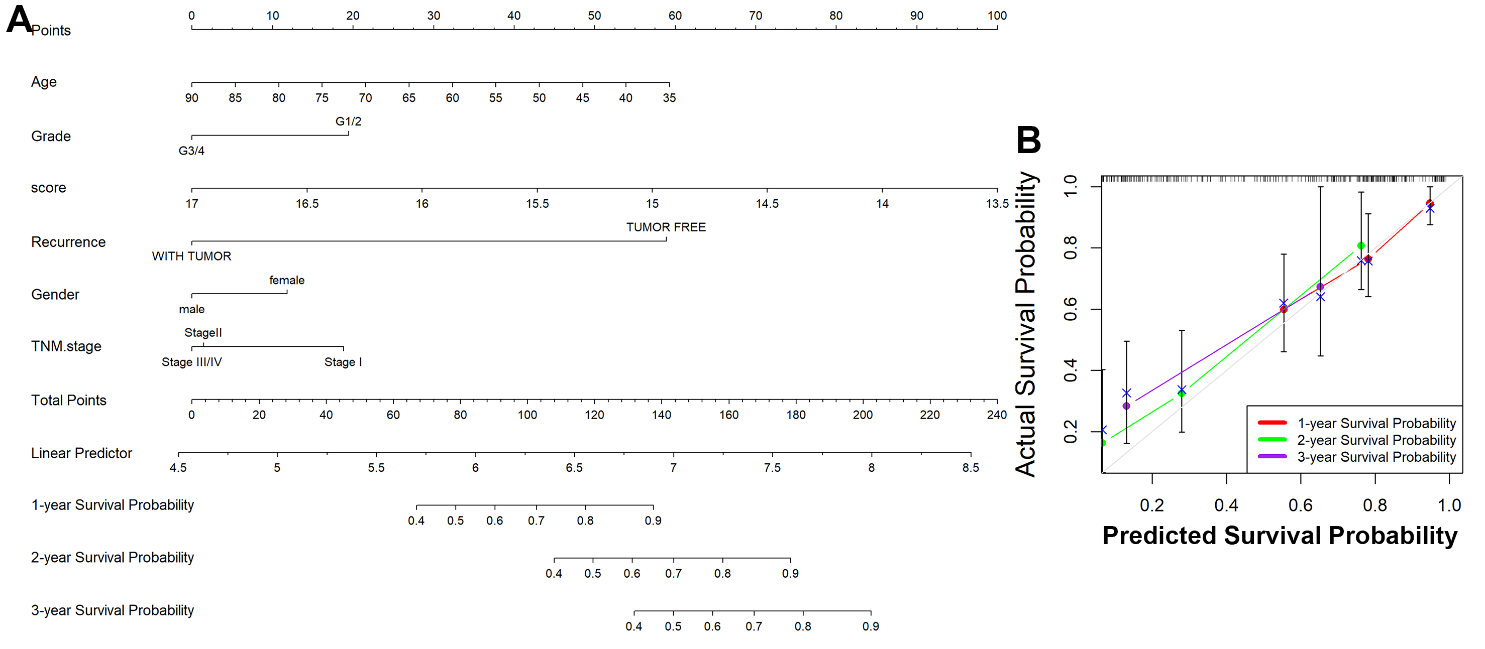


Figure S3 The predictive significance of the prognostic model was verified in the nomogram.


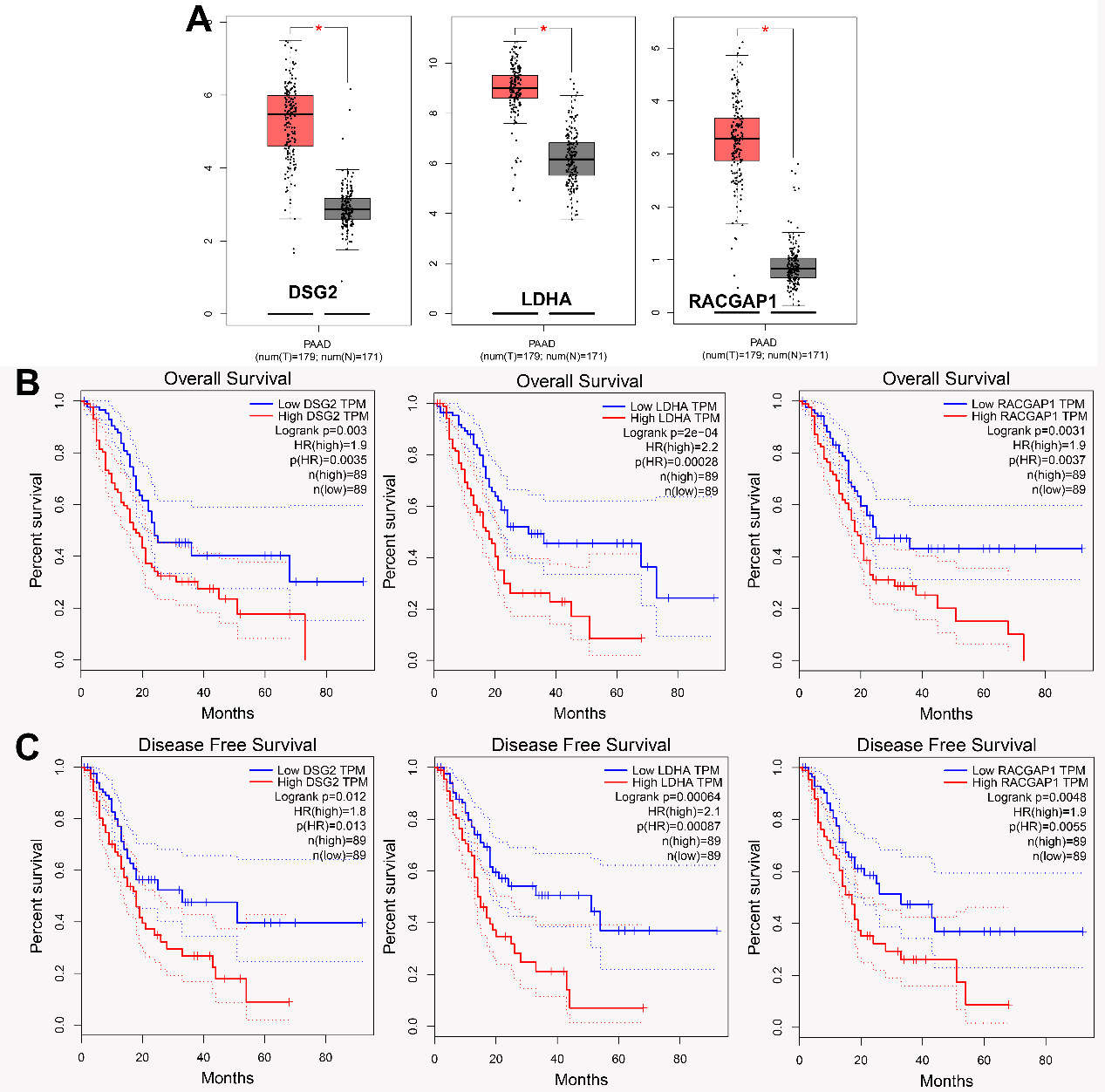


Figure S4 Expression and prognostic value of DSG2, LDHA, and RACGAP1 in the GEPIA database.


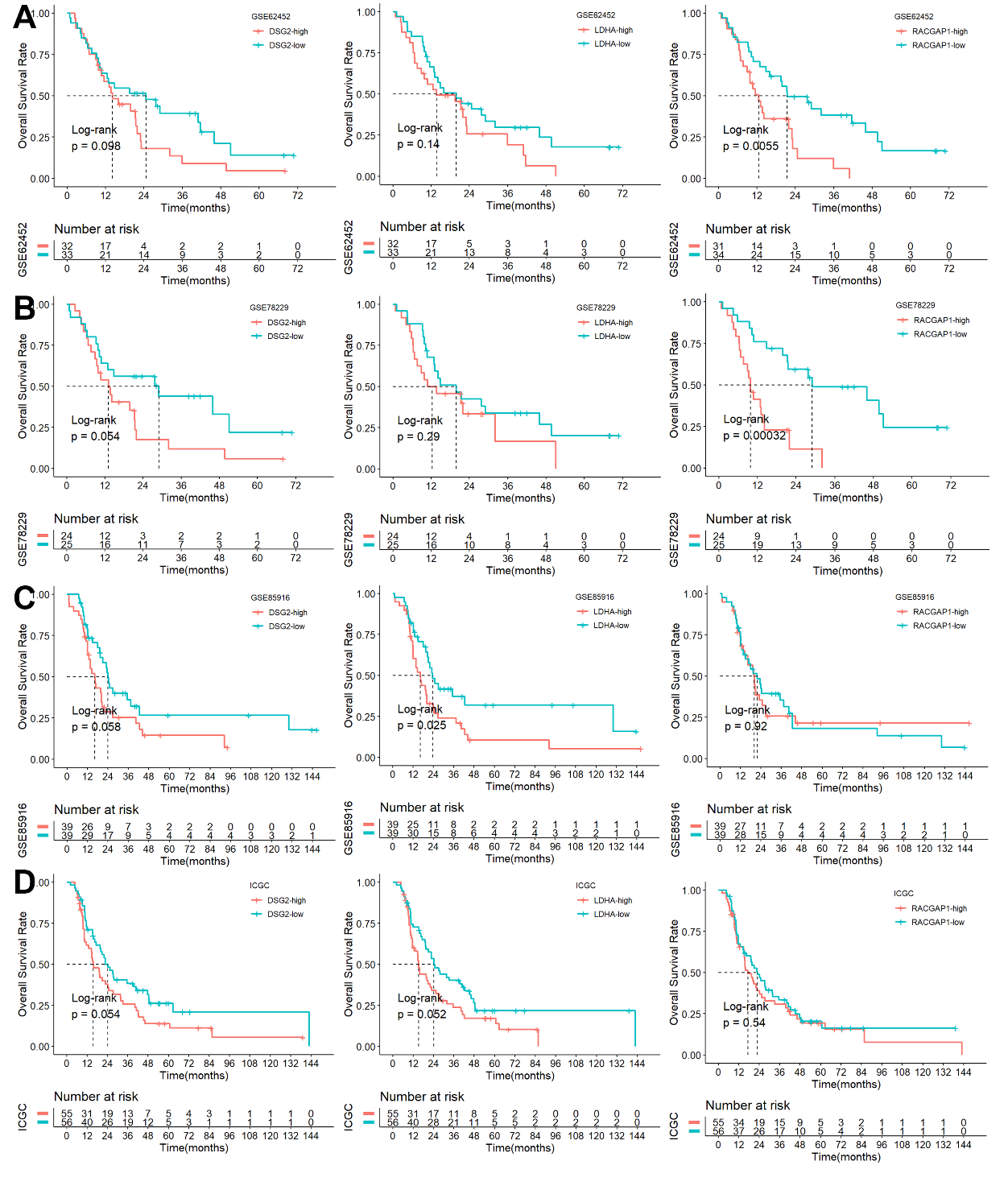


Figure S5 Prognostic value of DSG2, LDHA, and RACGAP1 in the (A) GSE62452, (B) GSE78229, (C) GSE85916 and (D) ICGC cohorts.


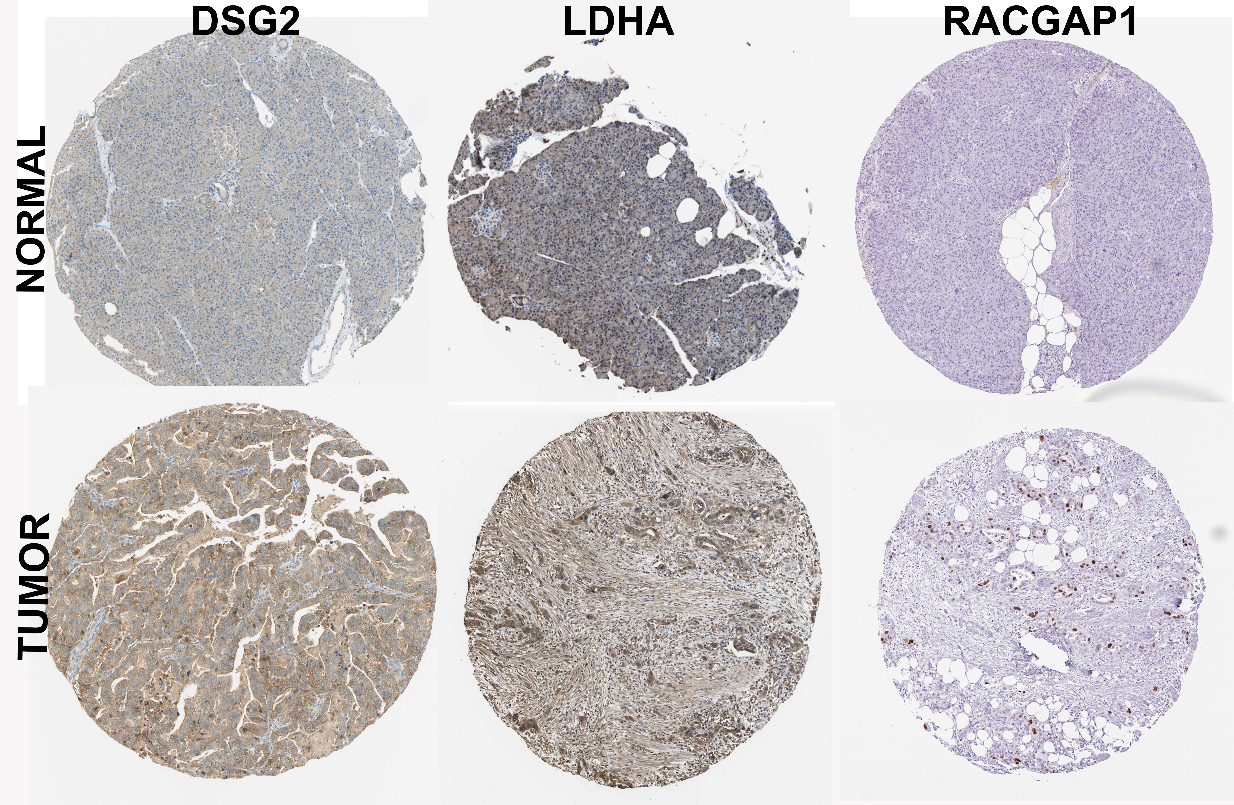


Figure S6 Expression and prognostic value of DSG2, LDHA, and RACGAP1 in the HPA databases.


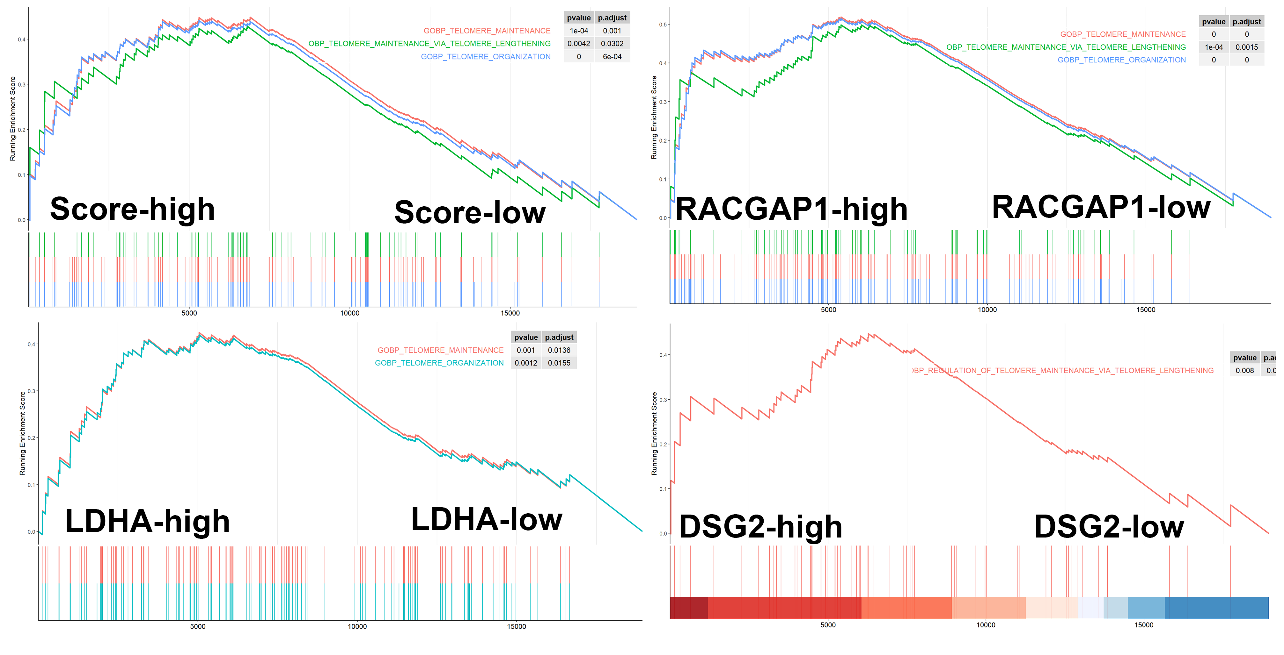


Figure S7 The relationship between score, DSG2, LDHA, RACGAP1, and telomere was analyzed by GSEA.

Table S1 Correlation of risk scores with genes associated with telomere-related genes

| Gene name | correlation coefficient | p-value | Gene name | correlation coefficient | p-value |
| --- | --- | --- | --- | --- | --- |
| RPAP3 | 0.361335 | 2.15E-06 | NAA35 | 0.420243 | 2.34E-08 |
| PRKAR2B | -0.3597 | 2.41E-06 | ANXA1 | 0.330008 | 1.69E-05 |
| ABCC8 | -0.31861 | 3.40E-05 | CAPRIN1 | 0.34214 | 7.82E-06 |
| BAIAP2L1 | 0.401449 | 1.09E-07 | MPHOSPH6 | 0.420058 | 2.38E-08 |
| PAFAH1B1 | -0.31496 | 4.22E-05 | IGF2BP3 | 0.465506 | 3.82E-10 |
| TEAD3 | 0.325669 | 2.21E-05 | RAC1 | 0.41796 | 2.84E-08 |
| E2F2 | 0.321693 | 2.82E-05 | HUS1 | 0.377687 | 6.69E-07 |
| MAP4K3 | 0.332988 | 1.40E-05 | BRIP1 | 0.557294 | 1.11E-14 |
| BRCA1 | 0.331486 | 1.54E-05 | CDK9 | -0.38829 | 3.03E-07 |
| ERCC1 | -0.32143 | 2.87E-05 | USP20 | -0.62309 | 6.59E-19 |
| FHL1 | -0.30883 | 6.05E-05 | DSCC1 | 0.384368 | 4.07E-07 |
| VRK2 | 0.437191 | 5.38E-09 | TFAP2A | 0.481879 | 7.39E-11 |
| HMGB3 | 0.448988 | 1.84E-09 | HMGA1 | 0.453496 | 1.21E-09 |
| ADSS | 0.465001 | 4.01E-10 | PLCB2 | -0.34755 | 5.48E-06 |
| RPL26L1 | 0.347931 | 5.34E-06 | CENPO | 0.385036 | 3.87E-07 |
| PI4K2B | 0.420624 | 2.27E-08 | DNA2 | 0.477079 | 1.21E-10 |
| LSG1 | 0.319437 | 3.23E-05 | BARD1 | 0.502629 | 8.10E-12 |
| PARP3 | 0.343107 | 7.34E-06 | OLA1 | 0.320399 | 3.05E-05 |
| LCP2 | -0.31307 | 4.72E-05 | ZGRF1 | 0.380188 | 5.56E-07 |
| CTNNA1 | 0.512925 | 2.55E-12 | SEC24B | 0.317594 | 3.61E-05 |
| DSG2 | 0.684651 | 7.02E-24 | BRCA2 | 0.466566 | 3.44E-10 |
| RFC2 | 0.318377 | 3.45E-05 | TMX1 | 0.32043 | 3.05E-05 |
| NFE2L3 | 0.502003 | 8.68E-12 | NAA30 | 0.345318 | 6.35E-06 |
| RAD51 | 0.576618 | 7.96E-16 | DNAJA4 | 0.381984 | 4.87E-07 |
| PIK3CB | 0.517269 | 1.55E-12 | PIF1 | 0.300109 | 9.94E-05 |
| THOC3 | 0.437149 | 5.40E-09 | FANCI | 0.548816 | 3.33E-14 |
| MXD1 | 0.300399 | 9.78E-05 | GCSH | 0.421898 | 2.03E-08 |
| SPA17 | 0.434031 | 7.12E-09 | TBCD | -0.30971 | 5.75E-05 |
| TAF2 | 0.385236 | 3.82E-07 | RPS11 | -0.32557 | 2.23E-05 |
| TNPO3 | 0.38283 | 4.57E-07 | HDGF | 0.369229 | 1.23E-06 |
| PRKCQ | -0.30366 | 8.13E-05 | RPS27A | -0.32281 | 2.64E-05 |
| LRRC40 | 0.30163 | 9.12E-05 | DUSP11 | 0.426201 | 1.41E-08 |
| PFKP | 0.618257 | 1.46E-18 | FANCD2 | 0.407165 | 6.89E-08 |
| PKM | 0.517905 | 1.44E-12 | ADPRH | -0.4011 | 1.12E-07 |
| PITX1 | 0.407889 | 6.50E-08 | CCNA2 | 0.713203 | 1.25E-26 |
| DNAJA2 | 0.320145 | 3.10E-05 | NAF1 | 0.309934 | 5.67E-05 |
| PABPC1 | 0.361203 | 2.17E-06 | TRIM7 | 0.309649 | 5.77E-05 |
| RAD18 | 0.545474 | 5.10E-14 | PRIM2 | 0.509243 | 3.88E-12 |
| TRIP13 | 0.585148 | 2.35E-16 | CCT6A | 0.340268 | 8.82E-06 |
| MBD3 | -0.34262 | 7.58E-06 | ZC3HAV1L | 0.444637 | 2.75E-09 |
| PDCD2 | -0.3596 | 2.42E-06 | DOK2 | -0.3765 | 7.30E-07 |
| HMMR | 0.761494 | 3.95E-32 | CHMP7 | -0.33048 | 1.64E-05 |
| MCM2 | 0.487471 | 4.13E-11 | TCF7L2 | 0.358293 | 2.65E-06 |
| IGF2BP2 | 0.578738 | 5.90E-16 | MKI67 | 0.651385 | 4.74E-21 |
| TSG101 | 0.334945 | 1.24E-05 | INCENP | 0.649087 | 7.22E-21 |
| ACTB | 0.348757 | 5.06E-06 | CHEK1 | 0.588727 | 1.40E-16 |
| ARHGAP15 | -0.508 | 4.46E-12 | PLCB3 | 0.321088 | 2.93E-05 |
| UBE2A | 0.333282 | 1.38E-05 | MRPL49 | 0.342532 | 7.62E-06 |
| TP73 | 0.493873 | 2.10E-11 | ALDOA | 0.554859 | 1.52E-14 |
| SENP1 | 0.391236 | 2.42E-07 | HMGA2 | 0.571745 | 1.57E-15 |
| HSP90AA1 | 0.45419 | 1.13E-09 | PDCD4 | -0.44065 | 3.94E-09 |
| NDC80 | 0.53059 | 3.22E-13 | DLG2 | -0.41851 | 2.71E-08 |
| TCF7 | -0.3613 | 2.15E-06 | CCT5 | 0.409138 | 5.88E-08 |
| BZW1 | 0.528368 | 4.20E-13 | DLAT | 0.426965 | 1.32E-08 |
| XPO1 | 0.347133 | 5.63E-06 | SLC7A11 | 0.36685 | 1.46E-06 |
| PALB2 | 0.552125 | 2.18E-14 | UEVLD | 0.478308 | 1.07E-10 |
| GRHL2 | 0.438228 | 4.90E-09 | CSNK1G3 | 0.420608 | 2.27E-08 |
| CHMP2B | 0.469754 | 2.52E-10 | NEK7 | 0.367708 | 1.37E-06 |
| RAB10 | 0.479027 | 9.90E-11 | NCAPD3 | 0.494198 | 2.02E-11 |
| ORC1 | 0.513931 | 2.28E-12 | ZNF827 | 0.303547 | 8.18E-05 |
| ATG16L1 | 0.400705 | 1.16E-07 | FLI1 | -0.39169 | 2.34E-07 |
| RAD54L | 0.606154 | 1.00E-17 | PDK1 | 0.464227 | 4.33E-10 |
| AURKA | 0.682913 | 1.01E-23 | SUV39H2 | 0.301416 | 9.23E-05 |
| PEBP1 | -0.48184 | 7.42E-11 | PLOD2 | 0.335331 | 1.21E-05 |
| GMIP | -0.31541 | 4.11E-05 | ANAPC1 | 0.337422 | 1.06E-05 |
| DHX32 | 0.334121 | 1.31E-05 | UCHL1 | -0.40967 | 5.63E-08 |
| RCOR1 | 0.601108 | 2.18E-17 | BUB3 | 0.326213 | 2.14E-05 |
| PABPC4 | -0.30767 | 6.46E-05 | EME1 | 0.533681 | 2.21E-13 |
| KIF4A | 0.686711 | 4.56E-24 | LSM11 | 0.318916 | 3.34E-05 |
| PUS7 | 0.399613 | 1.26E-07 | ADK | 0.355323 | 3.25E-06 |
| TF | -0.31557 | 4.07E-05 | SFR1 | 0.425982 | 1.43E-08 |
| ORC6 | 0.598383 | 3.31E-17 | HK1 | 0.387466 | 3.22E-07 |
| ESR1 | -0.34216 | 7.80E-06 | ATAD2 | 0.608394 | 7.05E-18 |
| TOX4 | 0.373411 | 9.13E-07 | NSMCE2 | 0.385625 | 3.71E-07 |
| TBX15 | 0.341298 | 8.25E-06 | SNX22 | -0.34241 | 7.68E-06 |
| CLSPN | 0.611429 | 4.37E-18 | RHBDL2 | 0.355042 | 3.31E-06 |
| CDC45 | 0.490537 | 2.99E-11 | HIST1H4H | 0.3849 | 3.91E-07 |
| CDC23 | 0.515652 | 1.87E-12 | TAGLN2 | 0.425282 | 1.52E-08 |
| MSH2 | 0.450299 | 1.63E-09 | CCAR2 | -0.30389 | 8.03E-05 |
| HSP90AB1 | 0.414659 | 3.73E-08 | PRMT2 | -0.36286 | 1.93E-06 |
| ERMP1 | 0.466162 | 3.58E-10 | RECQL4 | 0.321151 | 2.92E-05 |
| CIRBP | -0.64466 | 1.61E-20 | NAGS | 0.305923 | 7.15E-05 |
| HNRNPM | -0.33385 | 1.33E-05 | PLCD3 | 0.312061 | 5.01E-05 |
| PALM | -0.43071 | 9.52E-09 | RACGAP1 | 0.7723 | 1.52E-33 |
| PPM1F | -0.47895 | 9.98E-11 | RPL26 | -0.37497 | 8.16E-07 |
| TOP3B | 0.334331 | 1.29E-05 | TAL1 | -0.44964 | 1.73E-09 |
| DDX17 | -0.33931 | 9.39E-06 | ALPL | -0.37864 | 6.23E-07 |
| DMC1 | 0.389724 | 2.72E-07 | HAAO | -0.54611 | 4.71E-14 |
| CERK | -0.46239 | 5.17E-10 | DPY30 | 0.344643 | 6.64E-06 |
| PRMT5 | 0.476708 | 1.25E-10 | TDRD10 | -0.57091 | 1.76E-15 |
| POLE2 | 0.499529 | 1.14E-11 | PPM1K | -0.46666 | 3.41E-10 |
| AHSA1 | 0.359942 | 2.37E-06 | GMPS | 0.504854 | 6.33E-12 |
| SNW1 | 0.307587 | 6.50E-05 | TOPBP1 | 0.455143 | 1.03E-09 |
| VRK1 | 0.406573 | 7.23E-08 | RYK | 0.40374 | 9.08E-08 |
| YY1 | 0.325459 | 2.24E-05 | KLF15 | -0.39634 | 1.63E-07 |
| BRMS1L | 0.303247 | 8.32E-05 | RPN1 | 0.303111 | 8.39E-05 |
| NFATC2 | -0.46228 | 5.23E-10 | SENP2 | 0.405899 | 7.64E-08 |
| TTI1 | 0.410241 | 5.37E-08 | RFC4 | 0.433414 | 7.51E-09 |
| E2F1 | 0.450027 | 1.67E-09 | S100P | 0.310706 | 5.42E-05 |
| RPRD1B | 0.405996 | 7.58E-08 | CDC25A | 0.480103 | 8.87E-11 |
| RBBP8 | 0.359299 | 2.48E-06 | POC1A | 0.532749 | 2.48E-13 |
| CSTF2 | 0.530842 | 3.12E-13 | HMGB2 | 0.382577 | 4.66E-07 |
| PSMD10 | 0.470495 | 2.34E-10 | NAA15 | 0.42611 | 1.42E-08 |
| PGRMC1 | 0.350324 | 4.55E-06 | CHMP4C | 0.404759 | 8.37E-08 |
| POLA1 | 0.316348 | 3.89E-05 | RAD21 | 0.43814 | 4.94E-09 |
| PRPS2 | 0.351755 | 4.14E-06 | SUN1 | 0.394858 | 1.83E-07 |
| CDK16 | 0.321593 | 2.84E-05 | NOS3 | -0.30467 | 7.68E-05 |
| KLF5 | 0.495297 | 1.80E-11 | YWHAZ | 0.576422 | 8.18E-16 |
| PARP4 | 0.319725 | 3.18E-05 | SYK | -0.35605 | 3.09E-06 |
| ABCC1 | 0.300266 | 9.85E-05 | ARHGAP12 | 0.321047 | 2.93E-05 |
| IQCH | 0.321469 | 2.86E-05 | LRR1 | 0.363961 | 1.79E-06 |
| MCM4 | 0.583417 | 3.02E-16 | ARF6 | 0.436503 | 5.72E-09 |
| TUBB4A | -0.37576 | 7.70E-07 | ARHGAP42 | 0.450865 | 1.55E-09 |
| SNRNP70 | -0.34845 | 5.16E-06 | ARL5B | 0.334452 | 1.28E-05 |
| RNASEH2A | 0.36025 | 2.32E-06 | CCT2 | 0.332993 | 1.40E-05 |
| ASF1B | 0.553346 | 1.86E-14 | WEE1 | 0.326569 | 2.10E-05 |
| CCNE1 | 0.389227 | 2.82E-07 | PRKCB | -0.37923 | 5.97E-07 |
| USF2 | -0.51184 | 2.89E-12 | HSP90B1 | 0.336947 | 1.09E-05 |
| MET | 0.710788 | 2.21E-26 | TERF2IP | -0.39874 | 1.35E-07 |
| AIMP2 | 0.404034 | 8.87E-08 | PLK1 | 0.624016 | 5.65E-19 |
| RPA3 | 0.317034 | 3.73E-05 | VPS39 | -0.39333 | 2.06E-07 |
| EZH2 | 0.450297 | 1.63E-09 | NUDT21 | 0.354438 | 3.45E-06 |
| ANKMY2 | 0.320672 | 3.00E-05 | FBXO22 | 0.3063 | 6.99E-05 |
| ACTR3C | 0.30849 | 6.17E-05 | RRM1 | 0.532405 | 2.58E-13 |
| RARRES2 | -0.42867 | 1.14E-08 | ATG16L2 | -0.33321 | 1.38E-05 |
| NSMCE4A | -0.32333 | 2.56E-05 | SF1 | -0.446 | 2.42E-09 |
| ARHGAP21 | 0.356819 | 2.93E-06 | MAP4K2 | -0.31073 | 5.41E-05 |
| UBTF | -0.54898 | 3.27E-14 | ARF4 | 0.301493 | 9.19E-05 |
| PNPO | 0.350532 | 4.49E-06 | STIP1 | 0.418375 | 2.74E-08 |
| EZH1 | -0.35719 | 2.86E-06 | FEN1 | 0.402019 | 1.04E-07 |
| RPL34 | -0.41864 | 2.68E-08 | USP39 | 0.331368 | 1.55E-05 |
| KLF3 | 0.363003 | 1.91E-06 | MECP2 | -0.36952 | 1.21E-06 |
| NCAPG | 0.668264 | 1.93E-22 | UPF3A | -0.31778 | 3.57E-05 |
| PTPN5 | -0.35772 | 2.76E-06 | SLC25A6 | -0.38741 | 3.24E-07 |
| BIN2 | -0.39726 | 1.52E-07 | PGM2 | 0.433935 | 7.18E-09 |
| PTGES3 | 0.413389 | 4.15E-08 | CLIC3 | 0.322844 | 2.63E-05 |
| MAGOHB | 0.317082 | 3.72E-05 | ZNF32 | -0.42715 | 1.30E-08 |
| FOXM1 | 0.659077 | 1.13E-21 | YWHAG | 0.383941 | 4.21E-07 |
| RAD51AP1 | 0.668394 | 1.88E-22 | CDK1 | 0.675565 | 4.53E-23 |
| RFC5 | 0.491499 | 2.70E-11 | ELOVL6 | 0.423469 | 1.78E-08 |
| NUP107 | 0.467026 | 3.29E-10 | FOXN2 | 0.315916 | 3.99E-05 |
| GAPDH | 0.694292 | 9.00E-25 | PIK3CD | -0.38924 | 2.82E-07 |
| TPI1 | 0.551084 | 2.49E-14 | CD7 | -0.32147 | 2.86E-05 |
| ENO2 | 0.418625 | 2.68E-08 | EXO1 | 0.618726 | 1.35E-18 |
| SUDS3 | 0.351602 | 4.18E-06 | ATP2A2 | 0.353846 | 3.59E-06 |
| SRSF9 | 0.309024 | 5.98E-05 | BRSK2 | -0.32374 | 2.49E-05 |
| FANCE | 0.309037 | 5.97E-05 | ATR | 0.350645 | 4.46E-06 |
| E2F3 | 0.336587 | 1.12E-05 | CCNE2 | 0.5252 | 6.13E-13 |
| TFEB | -0.50264 | 8.09E-12 | LRRC25 | -0.31327 | 4.67E-05 |
| DUSP22 | -0.44365 | 3.00E-09 | CABP4 | 0.394442 | 1.89E-07 |
| MSH3 | 0.322711 | 2.65E-05 | RAB6A | 0.42513 | 1.54E-08 |
| LMNB1 | 0.588938 | 1.35E-16 | RMI2 | 0.438409 | 4.82E-09 |
| CSNK1A1 | 0.39853 | 1.37E-07 | RUVBL1 | 0.335688 | 1.18E-05 |
| ECT2 | 0.736248 | 4.20E-29 | YES1 | 0.413297 | 4.18E-08 |
| GBE1 | 0.471608 | 2.09E-10 | ATAD5 | 0.444803 | 2.70E-09 |
| NEK4 | 0.363876 | 1.80E-06 | HIC1 | -0.41638 | 3.24E-08 |
| DARS | 0.479124 | 9.80E-11 | CSTF2T | -0.35307 | 3.79E-06 |
| ORC2 | 0.305295 | 7.41E-05 | RPS27 | -0.35699 | 2.90E-06 |
| EPAS1 | -0.3567 | 2.96E-06 | DMAP1 | -0.40331 | 9.40E-08 |
| CACYBP | 0.332442 | 1.45E-05 | RMI1 | 0.370479 | 1.13E-06 |
| AMPD2 | -0.35717 | 2.86E-06 | CTC1 | -0.40595 | 7.61E-08 |
| SFPQ | -0.42124 | 2.15E-08 | AURKB | 0.459558 | 6.80E-10 |
| SRM | -0.31389 | 4.50E-05 | GATA2 | -0.36966 | 1.20E-06 |
| CDC20 | 0.577179 | 7.35E-16 | MED14 | 0.33269 | 1.43E-05 |
| DARS2 | 0.456857 | 8.79E-10 | RCC1 | 0.456209 | 9.35E-10 |
| NEK2 | 0.690558 | 2.01E-24 | CCDC43 | 0.431922 | 8.57E-09 |
| CENPF | 0.687657 | 3.73E-24 | HCLS1 | -0.46222 | 5.26E-10 |
| HMGN3 | -0.37091 | 1.09E-06 | ARHGAP45 | -0.35593 | 3.12E-06 |
| DCLRE1B | 0.403359 | 9.36E-08 | ZSCAN4 | 0.384613 | 4.00E-07 |
| GTF3C3 | 0.472494 | 1.92E-10 | MAPK15 | 0.311353 | 5.22E-05 |
| BCL11A | -0.37425 | 8.60E-07 | FANCB | 0.535144 | 1.85E-13 |
| MASTL | 0.53871 | 1.19E-13 | GINS3 | 0.462688 | 5.03E-10 |
| IQSEC3 | -0.35081 | 4.41E-06 | RAD51B | 0.427393 | 1.27E-08 |
| HSPH1 | 0.346831 | 5.75E-06 | KPNA2 | 0.393829 | 1.98E-07 |
| EPHX2 | -0.42839 | 1.17E-08 | FES | -0.43757 | 5.20E-09 |
| LYPLA1 | 0.44908 | 1.82E-09 | ANXA2 | 0.488987 | 3.52E-11 |
| TBX2 | -0.30498 | 7.54E-05 | EWSR1 | -0.37368 | 8.96E-07 |
| LRIF1 | 0.455901 | 9.63E-10 | AP2A2 | -0.38396 | 4.20E-07 |
| RPL21 | -0.39533 | 1.76E-07 | TSPYL2 | -0.46798 | 3.00E-10 |
| KIAA1191 | 0.351358 | 4.25E-06 | ADSSL1 | 0.337943 | 1.02E-05 |
| PMS2 | 0.343906 | 6.96E-06 | PRMT3 | 0.537682 | 1.35E-13 |
| CBX3 | 0.464621 | 4.17E-10 | IKZF1 | -0.42753 | 1.26E-08 |
| ATF1 | 0.332339 | 1.46E-05 | RPS23 | -0.33956 | 9.24E-06 |
| ARHGAP9 | -0.48773 | 4.02E-11 | FOXD2 | 0.325193 | 2.28E-05 |
| CDK2 | 0.545477 | 5.10E-14 | NAP1L1 | -0.30451 | 7.75E-05 |
| RAB38 | 0.3146 | 4.31E-05 | PTMA | 0.340548 | 8.67E-06 |
| DAW1 | 0.375307 | 7.96E-07 | TCEA1 | 0.326133 | 2.15E-05 |
| SRSF6 | -0.33737 | 1.06E-05 | NHEJ1 | 0.417598 | 2.92E-08 |
| GDAP1L1 | -0.36423 | 1.76E-06 | FANCA | 0.534178 | 2.08E-13 |
| CSE1L | 0.397696 | 1.47E-07 | FANCM | 0.390508 | 2.56E-07 |
| USP22 | -0.3056 | 7.28E-05 | TSPYL1 | -0.3897 | 2.72E-07 |
| PACSIN1 | -0.41216 | 4.59E-08 | PAX5 | -0.35589 | 3.13E-06 |
| RPS10 | -0.31187 | 5.06E-05 | EVL | -0.51892 | 1.28E-12 |
| GNMT | -0.30766 | 6.47E-05 | PIK3R4 | 0.355346 | 3.25E-06 |
| AHNAK | 0.322832 | 2.63E-05 | XRCC2 | 0.563595 | 4.78E-15 |
| FOSB | -0.33519 | 1.22E-05 | SLC39A10 | 0.313203 | 4.68E-05 |
| STAT5A | -0.41764 | 2.91E-08 | HIST1H3J | 0.306612 | 6.87E-05 |
| TRMT5 | 0.390034 | 2.65E-07 | C1D | 0.313087 | 4.72E-05 |
| PKMYT1 | 0.431078 | 9.22E-09 | RAD54B | 0.513003 | 2.53E-12 |
| PAICS | 0.529967 | 3.47E-13 | BLM | 0.37288 | 9.49E-07 |
| POT1 | 0.408845 | 6.02E-08 | PLCG2 | -0.41342 | 4.14E-08 |
| PHF20L1 | 0.321409 | 2.87E-05 | PRIM1 | 0.433102 | 7.72E-09 |
| PARP2 | 0.395029 | 1.80E-07 | WDHD1 | 0.65153 | 4.62E-21 |
| SGO1 | 0.651374 | 4.75E-21 | RPL10A | -0.3036 | 8.16E-05 |
| GAMT | -0.4023 | 1.02E-07 | OPA1 | 0.383882 | 4.22E-07 |
| JUND | -0.39452 | 1.88E-07 | TOP1 | 0.497089 | 1.48E-11 |
| CEP85 | 0.431211 | 9.12E-09 | MAP3K3 | -0.33909 | 9.52E-06 |
| POMT1 | -0.39093 | 2.48E-07 | PPP1R10 | -0.36196 | 2.06E-06 |
| EIF3G | -0.36355 | 1.84E-06 | GNL1 | -0.36613 | 1.54E-06 |
| PNCK | 0.461087 | 5.87E-10 | ZBTB48 | -0.32985 | 1.71E-05 |
| SLC7A10 | -0.46932 | 2.63E-10 | ARRDC5 | -0.33226 | 1.47E-05 |
| SYNE1 | -0.38662 | 3.44E-07 | DNAJC9 | 0.499422 | 1.15E-11 |
| NFATC1 | -0.47288 | 1.84E-10 | CLIC1 | 0.332121 | 1.48E-05 |
| TOP2A | 0.739623 | 1.74E-29 | REPIN1 | -0.34847 | 5.15E-06 |
| PIAS3 | 0.427473 | 1.26E-08 | PPME1 | 0.469793 | 2.51E-10 |
| RHPN2 | 0.399099 | 1.31E-07 | ZSWIM7 | -0.32207 | 2.76E-05 |
| PPARG | 0.403237 | 9.45E-08 | VAMP2 | -0.56432 | 4.33E-15 |
| PCNA | 0.347737 | 5.41E-06 | TRIM16 | 0.33741 | 1.06E-05 |
| RFC3 | 0.424566 | 1.62E-08 | DHFR | 0.481324 | 7.82E-11 |
| SLF1 | 0.362139 | 2.03E-06 | SETSIP | 0.38409 | 4.16E-07 |
| RRAS2 | 0.385973 | 3.61E-07 | YY2 | 0.409592 | 5.66E-08 |
| CCNB1 | 0.675932 | 4.20E-23 | RPS28 | -0.33812 | 1.01E-05 |
| CAMK1 | -0.32176 | 2.81E-05 | CEBPA | -0.33821 | 1.01E-05 |
| LDHA | 0.77703 | 3.47E-34 | DPP3 | 0.311493 | 5.18E-05 |
| CDC73 | 0.350297 | 4.56E-06 | RTEL1 | 0.384828 | 3.94E-07 |
| RPS15A | -0.36102 | 2.20E-06 | ANXA8L1 | 0.385792 | 3.66E-07 |
| DOCK2 | -0.32077 | 2.99E-05 | RPS10-NUDT3 | 0.318119 | 3.50E-05 |
| STK26 | 0.36868 | 1.28E-06 | HIST2H4A | 0.402823 | 9.77E-08 |
| CDCA8 | 0.623472 | 6.19E-19 | DOC2B | -0.42265 | 1.91E-08 |
| RPAP3 | 0.361335 | 2.15E-06 | HIST1H3H | 0.427942 | 1.21E-08 |

Table S2 Immune cells with differences in abundance between patients with higher or lower scores by the four algorithms

| Algorithms | Type of immune cells |
| --- | --- |
| CIBERSORT | naïve B cell, CD8 T cell, resting CD4 T cell, monocyte, and M2 macrophage |
| TIMER | CD4 T cells |
| xCELL | B cell, naïve CD4 T cell, CD8 T cell, central memory CD8 T cell, class-switched memory B cell, common lymphoid progenitor, myeloid dendritic cell, endothelial cell, cancer-associated fibroblast, hematopoietic stem cell, macrophage, macrophage M1, macrophage M2, memory B cell, monocyte, NK T cell, Th1 CD4 T cell, and activated myeloid dendritic cell |
| MCPcounter | T cell, CD8 T cell, B cell, monocyte, endothelial cell, myeloid dendritic cell, and macrophage monocyte |

Table S3 18 Tumor-sensitive drugs telomere-related genes

| Drug | correlation coefficient | p-value |
| --- | --- | --- |
| aldoxorubicin | -0.42796 | 0.000722 |
| Epirubicin | -0.40255 | 0.001574 |
| Actinomycin D | -0.38882 | 0.002339 |
| AT-7519 | -0.38839 | 0.002368 |
| AZD-1208 | -0.38599 | 0.002533 |
| Doxorubicin | -0.37944 | 0.003038 |
| Elliptinium Acetate | -0.36577 | 0.004388 |
| 6-Thioguanine | 0.360334 | 0.005057 |
| Allopurinol | 0.356693 | 0.005553 |
| BN-2629 | -0.35614 | 0.005632 |
| Pipamperone | -0.35421 | 0.005917 |
| Daunorubicin | -0.34914 | 0.006722 |
| Homoharringtonine | -0.34626 | 0.007223 |
| BPTES | -0.34578 | 0.007309 |
| Vinblastine | -0.34373 | 0.007687 |
| Teniposide | -0.34092 | 0.008234 |
| Paclitaxel | -0.33581 | 0.009318 |
| DAUNORUBICIN | -0.33517 | 0.00946 |
